# Comparative Genomic Analysis Reveals Genetic Variations in Multiple Primary Esophageal Squamous Cell Carcinoma of Chinese Population

**DOI:** 10.3389/fonc.2022.868301

**Published:** 2022-04-20

**Authors:** Jinxiao Liang, Yinjie Wang, Lei Cai, Jinshi Liu, Junrong Yan, Xin Chen, Xiaoying Wu, Qixun Chen

**Affiliations:** ^1^ Department of Oncological Surgery, Cancer Hospital of the University of Chinese Academy of Sciences (Zhejiang Cancer Hospital), Hangzhou, China; ^2^ Institute of Cancer and Basic Medicine (IBMC), Chinese Academy of Sciences, Hangzhou, China; ^3^ Geneseeq Research Institute, Nanjing Geneseeq Technology Inc., Nanjing, China

**Keywords:** esophageal squamous cell carcinoma, physiologically normal mucosa, multiple primary cancer, whole-exome sequencing, genetic analysis

## Abstract

Esophageal squamous cell carcinoma (ESCC) is one of the most common and lethal malignant tumors. The incidence of malignant transformation of esophageal mucosa increases greatly due to long-term exposure to factors such as smoking, drinking, and poor eating habits. Furthermore, multiple primary tumors could occur synchronously or asynchronously in the upper aerodigestive tract, especially in the esophagus, adding difficulty to the treatment of ESCC. Genetic mutations are important during the malignant transformation from normal mucosa to esophageal cancer, but the underlying mechanism has not been fully elucidated. In this study, we used whole-exome sequencing (WES) to profile genetic variations in physiologically normal mucosa (PNM) and ESCC tumors, as well as PNM of non-ESCC subjects. We found significant differences in mutation frequencies of *NOTCH1* and *NOTCH2*, copy number variations (CNVs) at both gene and chromosomal arm levels, and cancer-related HIPPO, WNT, and NRF2 signaling pathways between ESCC tumors and normal mucosa. Our analysis of both primary tumors and paired PNM in bifocal ESCC revealed three different primary tumor evolution modes, and the most common mode exhibited a complete genomic divergence in all the samples from the same patient. Furthermore, the mutation frequency of *TP53* was significantly higher in ESCC cases than that in non-ESCC cases. Overall, our results provide important evidence for further elucidating the mechanisms of genetic mutations underlying the cause of ESCC.

## Introduction

Esophageal cancer was the seventh most common cancer and the sixth leading cause of cancer-related death worldwide, causing approximately 540,000 deaths in 2020 globally (1). Esophageal squamous cell carcinoma (ESCC) is the dominant subtype of esophageal cancer worldwide, especially prevalent in China (1). The incidence and mortality of ESCC in China accounted for half of the total cases in the world by 2016 (2). Due to its insidious onset and early lymphatic metastasis, the diagnosis of ESCC was usually delayed, resulting in a low five-year survival rate of 15-20% (3). Therefore, ESCC has become a major health challenge for the local community.

Carcinogenesis is a long-term process in which many precancerous cellular clones harboring mutations in known cancer-related genes can exist independently in the physiologically normal tissues before cancer development (4). External environment and genetic factors can alter the gene expression of these clones and affect their expansion, resulting in the formation of heterogeneous tumor cell populations and progression into cancer. Due to the long-term exposure to risk factors such as drinking and smoking, the upper aerodigestive tract mucosa of the esophagus, head, and neck, etc, can form “field cancerization” and further develop into squamous cell carcinoma synchronously or asynchronously (5). This process could also take place in the other upper aerodigestive tract mucosa after the esophageal cancer operation and affects the prognosis of patients (6). Long-term exposure could also result in multiple lesions, as two or more malignant lesions can be observed during the dissection of postsurgical esophageal cancer specimens. Since these esophageal lesions are sometimes physically distant from each other, it is not clear whether these lesions are related. Moreover, the relationships between the lesions and normal esophageal mucosa are still elusive.

In this study, we collected tumor and physiologically normal mucosa (PNM) samples from ESCC patients with single or multiple primary tumors, as well as PNM samples of non-ESCC gastric cancer (GC) participants. The specimens were subject to molecular profiling using whole-exome sequencing (WES). We further performed comprehensive comparisons across the tissues to understand the underlying genetic changes and potential interfocal relationships in the primary tumors of ESCC patients.

## Patients and Methods

### Patients

A total of 13 ESCC patients from Zhejiang Province, China, after esophagectomy were enrolled, in which 8 cases had two primary lesions and 5 cases had a single lesion. Fresh tumor tissues and PNM were obtained during the surgical operation. Totally 21 tumor and 13 PNM samples of ESCC were collected. We resected the PNM samples from the upper or lower part of the esophageal mucosa when the primary lesions were present in the lower or upper esophagus. Furthermore, the distance between each PNM and its closest tumor was more than 5 cm. Besides, we collected esophageal PNM samples from five patients with gastric cancer (GC) undergoing total gastrectomy. All their PNM samples were collected in the upper esophagus from > 10 cm far away from the GC lesions, and no lesion in the esophagus was found in these five GC patients. All cases were operated on in the Cancer Hospital of the University of Chinese Academy of Sciences (Zhejiang Cancer Hospital) from April 2019 to February 2021. Diagnosis, validity, and tumor purity of the specimens were confirmed by two independent pathologists of Zhejiang Cancer Hospital. In addition, 5 ml peripheral blood was collected from each patient and placed into EDTA-coated tubes (BD Biosciences). White blood cells (WBCs) were extracted as the control to determine germline variations, and our study was focused on somatic alterations. This study was approved by the ethics committee of Zhejiang Cancer Hospital (Approval No. IRB-2022-154). All participants were informed and consent to sample collection, intended research, and publication usage. Written consent was collected according to the ethical regulations of Zhejiang Cancer Hospital. The next-generation sequencing (NGS) was performed in a Clinical Laboratory Improvement Amendments (CLIA)- certified and College of American Pathologists (CAP)- accredited clinical testing laboratory (Nanjing Geneseeq Technology Inc., China). All samples were shipped to the clinical testing laboratory following the required conditions.

### DNA Extraction and Quantification

Genomic DNA from fresh tumor tissue, PNM, and WBCs was extracted using DNeasy Blood & Tissue kit (Qiagen) following the manufacturer’s instruction. Purified genomic DNA was qualified by Nanodrop2000 for A260/280 and A260/A230 ratios (Thermo Fisher Scientific). All DNA samples were quantified by Qubit 3.0 using the dsDNA HS Assay Kit (Life Technologies) according to the manufacturer’s recommendations.

### Whole Exome Sequencing (WES) and Data Processing

For WES library construction, we fragmented 2μg DNA using Covaris M220 sonication system (Covaris), followed by end-repairing, A-tailing, and adaptor ligation and purification by KAPA Hyper Prep Kit (KAPA Biosystems). The resultant libraries were amplified and purified before exome capture using the xGen Exome Research Panel v1.0 (Integrated DNA Technologies). The enriched libraries were then sequenced using the Illumina HiSeq 4000 platform with 2×150 bp pair-end reads. The mean raw coverage depth was ~60× for the WBC samples and ~200× for the tumor and PNM samples.

Paired-end sequencing data were aligned to the reference human genome (build hg19) with the Burrows-Wheeler Aligner (bwa-mem) (7). Alignment results (BAM files) were further processed for de-duplication, base quality recalibration, and indel realignment using the Picard suite (http://picard.sourceforge.net/) and the Genome Analysis Toolkit (GATK) (8). MuTect with default parameters was applied to the paired PNM and tumor BAM files to identify somatic single nucleotide variants (SNVs) (9). SNVs in the 1000 Genomes project and dbSNP with frequency >1% were excluded. Small insertions and deletions (indels) were detected using SCALPEL (10). SNV and indel annotation was performed by ANNOVAR using the hg19 reference genome and 2014 versions of standard databases and functional prediction programs (11). Gene-level copy number ratios were calculated by CNVKit using the CNVKit algorithm, relative copy-ratios for each exon were calculated by correcting for imbalanced library size, GC bias, sequence repeats, and target density. The log2 ratio values of 2.0 and 0.6 were used as the cut-off for copy number gain and copy number loss of tissue samples, respectively. Chromosome arm-level somatic copy number variations (CNVs) were analyzed by FACETS with a 0.2 drift cut-off for unstable joint segments. Chromosome instability score (CIS) was defined as the proportion of the genome with aberrant (purity-adjusted segment-level copy number >=3 or <=1) segmented copy number. Mutational signature enrichment weights were calculated using the sigminer R package (12). Treeomics (13) was used to reconstruct the phylogenetic relationships with maximum likelihood. Different tissues that were grouped together into the same clades were determined as convergent, otherwise, they were considered as divergent. The mutant-allele tumor heterogeneity (MATH) analysis was performed using inferHeterogeneity in the Maftools package (14).

### Statistical Analyses

Quantitative data were displayed as the median value (range) or the number of patients (percentage). Comparisons of proportion between two groups were done using Fisher’s exact test. Wilcoxon rank-sum test was performed to compare the mutation number, CIS, and MATH between different groups. Differences in mutation number among PNM of ESCC, PNM of GC, and tumor of ESCC were analyzed using the Kruskal-Wallis test. A two-sided P value of less than 0.05 was considered significant for all tests unless indicated otherwise. All statistical analyses were done in R (v.3.6.0).

## Results

### Baseline Characteristics of Enrolled Patients

As demonstrated in **Figure S1**, we enrolled 13 ESCC patients (double-primary: 8; single-primary: 5) whose tumor and PNM samples were collected to perform WES analysis. Five GC patients were also enrolled whose PNM samples were collected and subject to WES. Participants’ demographics and clinical characteristics are listed in **Tables 1** and **S1**. The median age of the 13 ESCC patients was 66 years. 12 of them were male. 7, 2, 10, and 11 of the patients had hypertension history, ESCC family history, smoking, and drinking history, respectively. The study included Stage I, II, and III patients (1, 4, and 8 cases, respectively). For tumor differentiation level, 3 of the ESCC patients were defined as high differentiation, 4 were moderate, and the other 6 were low (**Table 1**). All the GC participants were male, with a median age of 71 years (**Table S1**).

**Table 1:** Baseline characteristics of ESCC patients enrolled in this study.

Variables	Number (%)
Total	13 (100%)
Median age, years (range)	66 (49-75)
Gender	
Male	12 (92.3%)
Female	1 (7.7%)
Hypertension history	
Yes	7 (53.8%)
No	6 (46.2%)
ESCC family history	
Yes	2 (15.4%)
No	11 (84.6%)
Smoking history	
Yes	10 (76.9%)
No	3 (23.1%)
Drinking history	
Yes	11 (84.6%)
No	2 (15.4%)
Tumor lesion	
Single primary lesion	5 (38.5%)
Double primary lesion	8 (61.5%)
Tumor differentiation	
high	3 (23.1%)
moderate	4 (30.8%)
low	6 (46.2%)
T stage	
T1	3 (23.1%)
T2	3 (23.1%)
T3	7 (53.8%)
N stage	
N0	4 (30.8%)
N1	5 (38.5%)
N2	4 (30.8%)
TNM stage	
I	1 (7.7%)
II	4 (30.8%)
III	8 (61.5%)

### Comparison of Genomic Alterations Between Tumor and PNM in Patients With ESCC

**Figure 1:**
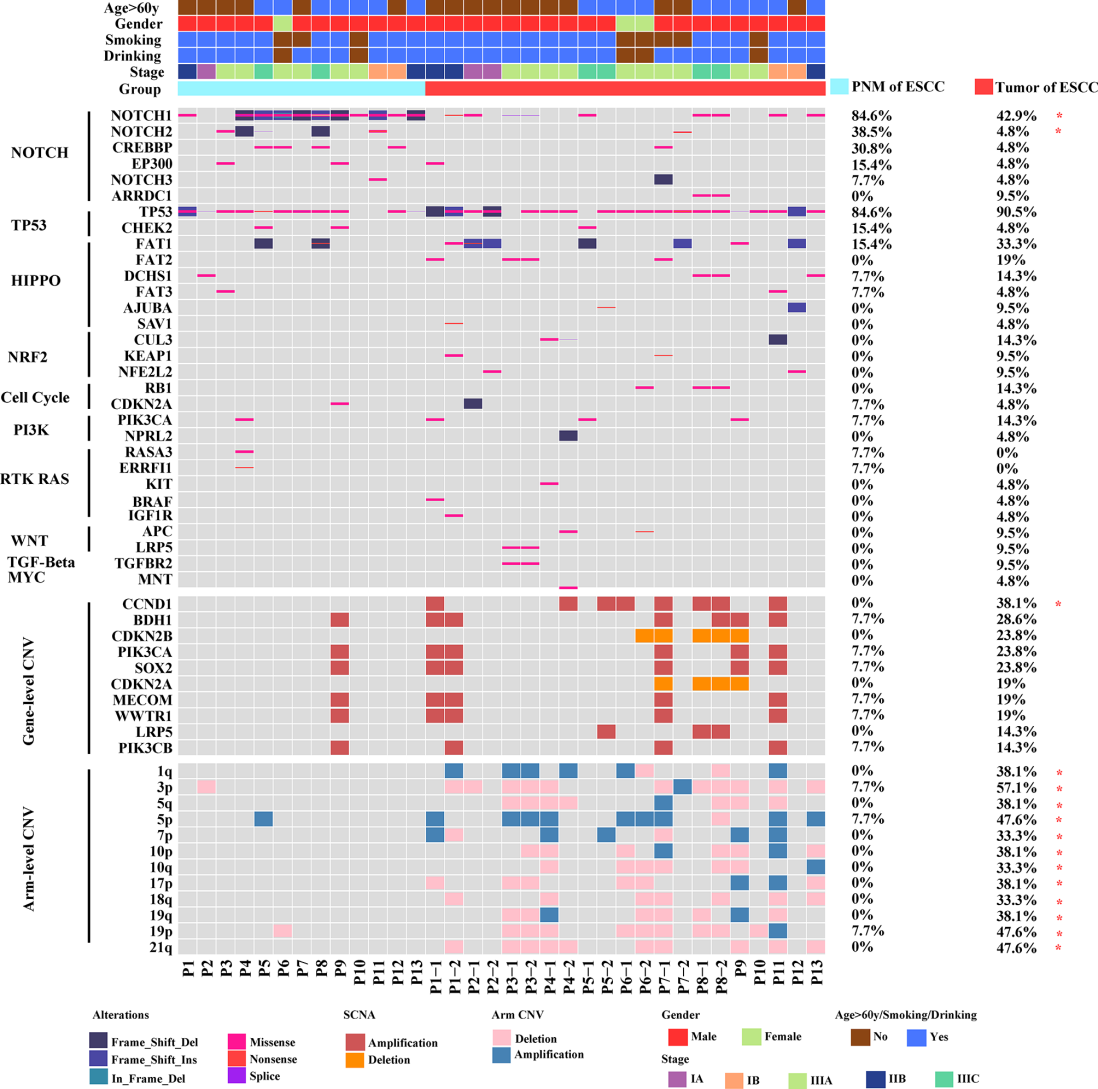
Mutational landscapes of ESCC PNM and tumor samples by whole-exome sequencing. The age, gender, smoking, drinking, stage (determined according to the 8^th^ Edition American Joint Committee on Cancer (AJCC) staging system for esophageal cancer), and group information are listed for each sample. In the mutational landscapes, top panel: SNV type, related signaling pathway, and frequencies in ESCC PNM and tumor; middle panel: gene copy number variations and their frequencies in ESCC PNM and tumor; bottom panel: chromosomal arm level copy number variations and their frequencies in ESCC PNM and tumor (*: statistically significant).

To understand the molecular characteristics underlying ESCC, we set out mutational profiling using tumor and normal mucosa tissues from ESCC patients. The WES results in **Figure 1** outlined the key differences in genomic alterations between tumor and normal mucosa. We did not observe significant enrichment of any somatically mutated gene in the tumor sample. Conversely, the mutation frequencies of *NOTCH1* and *NOTCH2* are significantly higher in PNM (**Figure 1**, top panel). The frequency of copy number changes was also examined, and we found a significant increase associated with *CCND1* in tumors (**Figure 1**, middle panel). In all the chromosomal arms, the frequency of copy number variation (CNV) in tumors was remarkably higher than that in PNM (**Figure 1**, bottom panel).

**Figure 2:**
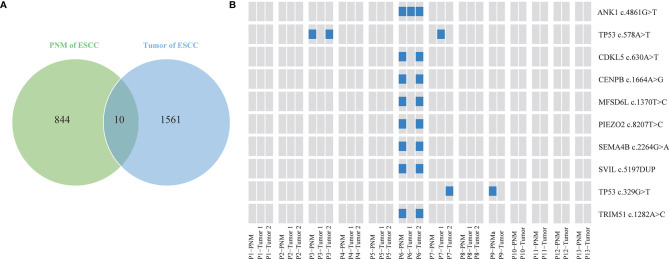
Distribution of the ESCC somatic single nucleotide variants. **(A)** Venn diagram showing the numbers of unique and shared somatic mutations in ESCC PNM and tumor samples. **(B)** Schematic diagram showing the distribution of shared mutations by ESCC tumor and PNM in specific patients. The presence of each shared mutation is indicated by the blue box.

**Figure 3:**
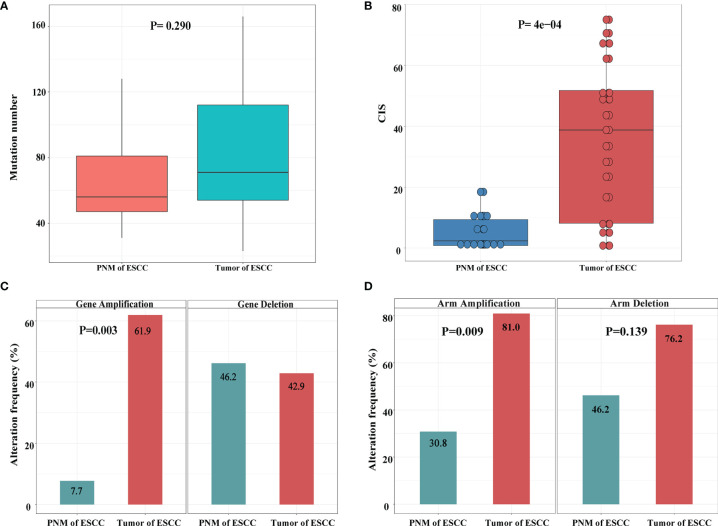
Summary of genomic variations in the ESCC samples. **(A)** Comparison of mutation numbers per sample in ESCC PNM and tumor. **(B)** Comparison of CIS between ESCC PNM and tumor samples. Comparisons of **(C)** gene level and **(D)** chromosomal arm level copy number amplification (left) and deletion (right) between ESCC PNM and tumor samples.

We further investigated the distribution of somatic gene mutations in ESCC tumors and PNM. In total, we found 854 mutations in the esophageal PNM and 1571 mutations in the tumors (**Figure 2A**). The median number of mutations in tumors (71, range 23-166) is higher than that in PNM (56, range 31-128) but lacks statistical significance (p=0.290, Wilcoxon test) (**Figure 3A**). Ten of the somatic mutations are found to present in both tumor and PNM, while 8 of them are actually from Patient P6 (**Figures 2A, B**). Detailed information about these tumor-PNM shared mutations was summarized in **Table S2**. In addition, we scrutinized the somatic mutations for their base mutation patterns, mutation types, and functional outcomes (inactivation vs. non-inactivation) and found high-degree similarity between tumor and PNM from ESCC (**Figure S2**). The somatic mutation pattern was also analyzed for the NOTCH genes, which are more frequently mutated in PNM (**Figures 1** and **S3**). There were many code-shifting mutations of *NOTCH1* in PNM from the mutation type, but no significant difference. The mutation frequency of *NOTCH2* gene in PNM was significantly higher than that in the tumor. However, there was no significant difference between the mutation types.

The genome stability conditions varied between the tumor and PNM samples. We calculated the chromosome instability score (CIS) of tumor and PNM, and the scores in the tumors are significantly higher than those in PNM (**Figure 3B**). The CNV analysis revealed significantly higher incidences of copy number amplification in the tumor than in PNM at both gene and chromosomal arm levels (P= 0.003 and 0.009, respectively, Fisher’s exact test, **Figures 3C, D**). As for the incidence of copy number deletion, tumor and PNM samples are similar at the gene level (**Figure 3C**). The incidence of chromosomal arm level copy number deletion in the tumor is moderately higher than that in PNM (P=0.139, Fisher’s exact test, **Figure 3D**).

### Changes in Signaling Pathways Between ESCC Tumors and PNM

**Figure 4:**
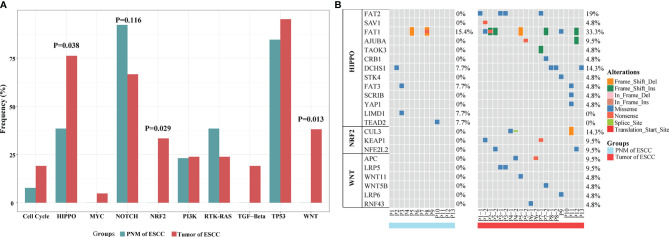
Signaling pathway analysis of mutations in ESCC PNM and tumor. **(A)** Bar graphs comparing the proportions of patients carrying mutations in the signaling pathway-related genes between ESCC PNM and tumor. Note that the frequencies of the HIPPO, NRF2, and WNT signaling pathways are significantly different between the PNM and tumor. **(B)** Details of mutation type and distribution in the genes related to the HIPPO, NRF2, and WNT signaling pathways.

For signaling pathway analysis, we classified key cancer-associated genes into ten canonical mitogenic signaling pathways (15). Then, we counted the number of samples that have at least one gene mutated in each pathway and computed the proportion of total samples altered in each pathway. Compared with normal mucosa, ESCC tumors exhibited significantly higher mutation frequencies in several cancer-related pathways, including the HIPPO (P=0.038, Fisher’s exact test), WNT (P=0.013, Fisher’s exact test), and NRF2 (P=0.029, Fisher’s exact test) pathways (**Figure 4A**). The NOTCH pathway is highly mutated in PNM relative to the tumor but lacks statistical significance (**Figure 4A**). Additionally, no somatic mutation was detected in genes of the NRF2 or WNT pathway in PNM (**Figure 4B**), but these signaling pathways are frequently mutated in the tumor. We also performed mutation signature analysis (**Figure S4**) and found that the Age signature (SBS1) is highly prevalent in both PNM and tumor (16). The enrichment of APOBEC signature (SBS2) in the tumor is significantly higher than that in PNM (P=0.021, Wilcoxon test). The signatures of Ultraviolet (SBS7, P=0.081, Wilcoxon test), POLE (SBS10a/b, P=0.092, Wilcoxon test), MMRdeficiency (SBS15, P=0.550, Wilcoxon test), and BRCA (SBS3, P=0.630, Wilcoxon test) in the tumor are also more enriched but lack statistical significance.

### Phylogenetic Analysis of Patients With Multiple Primary Esophageal Tumors

**Figure 5:**
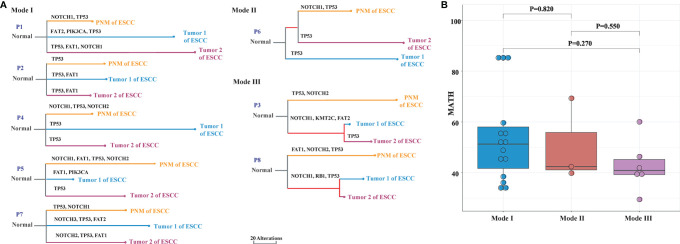
Phylogenetic analysis of ESCC PNM and tumor in multiple primary ESCC. **(A)** Phylogenetic trees demonstrating the three primary tumor evolutionary modes between primary tumors and corresponding PNM. The colors of lines and nodes denote different tissue samples. The branch length is in the scale of alterations with the scale bar indicating 20 alterations. **(B)** The MATH scores of each tissue sample that are grouped by evolutionary modes.

We performed phylogenetic analyses of somatic SNVs on specimens from 8 ESCC patients with multiple primary tumors to examine their interfocal heterogeneity, as well as the relationships between tumor and normal mucosa. Based on the genomic similarity among the two primary tumors and PNM of the same case, the genetic divergence patterns of the 8 patients could be categorized into three modes. In Mode I, the PNM and two tumor samples are mutually divergent and distribute at different clades. In Mode II, one of the two tumor samples shows convergence with PNM, and they cluster in the same clade. In Mode III, the two tumor samples but no PNM have convergence and cluster in the same clade. We found 5 patients in Mode I, 1 patient in Mode II, and 2 patients in Mode III (**Figure 5A**).

For every tumor and PNM sample, the MATH score was calculated, grouped into the aforementioned three modes, and compared to evaluate the heterogeneity of the different modes. We observed a gradual downward trend of the MATH scores from Mode I to Modes II and III (**Figure 5B**), consistent with the patterns shown in the hierarchical clustering dendrograms (**Figure 5A**).

### Genome Differences Between ESCC and Non-ESCC Subjects

To explore the unique genomic alterations in ESCC, we incorporated genomic analysis on esophageal PNM of GC patients and made the comparison with ESCC tissue samples. Remarkably, there is a significant difference in the prevalence of mutations in the *TP53* gene (**Figures 1** and **S5**), as this gene is commonly mutated in both the tumor and PNM samples of ESCC but not mutated in the GC samples (p=0.0003, P=0.002). The detailed *TP53* somatic mutation information is listed in **Table S3**. We observed that the most frequent *TP53* single base substitution (SBS) is the G:C>T:A transversion (26.2%) related to smoking in ESCC, followed by the G:C>A:T transition (19.0%) probably associated with chronic inflammation and the aristolochic acid-related A:T>T:A transversion (19.0%) (17). The genomic profiling of GC PNM also identified alterations in other cancer-related genes, including *NOTCH1*, *NOTCH2*, *NF1*, and *STK11* (**Figure S5A**). The median number of mutations per sample was 15 (5–62) in PNM of GC, 56 (31–128) in PNM of ESCC patients, and 71 (23–166) in the tumor of ESCC (**Figure S5B**). The number of mutations in PNM of GC was significantly lower than that in ESCC tumor (P=0.021, Wilcoxon test) and was also lower than that in ESCC PNM but not significant (P=0.100, Wilcoxon test). Based on the signaling pathway analysis comparing the ESCC and GC samples, we noticed that tumor and PNM of ESCC patients had higher frequencies of mutations in cancer-related signaling pathways in general (**Figure S5C**). Specifically, mutations are significantly enriched in the tumor and PNM of ESCC patients than that of GC PNM (P=0.002 and 0.022, respectively, Fisher’s exact test) for the *TP53* signaling pathway. The comparison of mutation signatures is shown in **Figure S6**, showing no significant difference between ESCC and GC patients in the prevalent mutation signatures.

## Discussion

The progressive accumulation of spontaneous mutations in human cells throughout life could cause cancer (18). Studies have shown that esophageal epithelial cells can accumulate somatic mutations with age, and this process is associated with the development of esophageal cancer, one of the most common and deadliest cancer types (4, 19). However, the role of somatic mutations in the pathological process of normal cells evolving into esophageal cancer has not been fully understood. In this study, we performed WES on both esophageal tumors and normal esophageal mucosa and set out a comprehensive comparison on their mutational landscapes.

Our comparative analysis identified the genomic alterations in tumor and PNM of ESCC patients. Previous studies have reported a significantly higher frequency of *NOTCH1*, *NOTCH2*, and other NOTCH gene mutations in aged (≥50 years) normal esophageal tissues compared with ESCCs (4, 19). These studies further proposed that ESCCs are more likely to evolve from esophageal epithelium without NOTCH mutations caused by the effects of lifestyle risk. In accordance with that, we showed the mutation frequencies of *NOTCH1* and *NOTCH2* genes in esophageal mucosa of our patients (median age: 66 years, range: 49-75 years), most of whom have smoking and drinking history, were significantly higher than those in esophageal carcinoma. As NOTCH signaling promotes keratinocytes differentiation, the *NOTCH1* and *NOTCH2* mutations may confer a competitive advantage in normal esophageal epithelium by tilting cell fate balance away from differentiation toward proliferation (20, 21). We also found that the copy number amplification of *CCND1* in esophageal cancer was significantly more frequent than that in esophageal mucosa, consistent with the notion that *CCND1* amplification is a common genetic aberration in ESCC and may promote tumor cell proliferation (22, 23). Esophageal cancer is characterized by frequent copy number changes (19), which tend to cause genetic variations and are closely related to the occurrence and development of cancer (24–26). We examined the level of copy number variations at both gene and chromosomal arm levels and found significantly higher frequencies of copy number amplification at both levels in ESCC. Additionally, copy number deletion at the chromosomal arm level also occurs more frequently in the tumor of ESCC than in PNM but lacks statistical significance. Based on our chromosomal stability assessment, ESCC tumors exhibited a significantly higher level of chromosomal instability than normal esophageal mucosa, which could be implicated in the metastasis, prognosis, and treatment efficacy of ESCC patients (27, 28).

Our genome-wide analysis also revealed the distinct landscapes of somatic mutations between the ESCC tumors and PNM. Among the total of 2,415 mutations we detected, 1,571 are in esophageal cancer and 854 are in normal esophageal mucosa. Only 10 of them are shared by esophageal cancer and esophageal mucosa. Furthermore, within the 10 shared mutations, 9 were present in the pair of tumor and PNM from the same patients, while 8 of them are from Case P6. This finding suggested that esophageal cancer and aged esophageal mucosa rarely share the same mutations. In the meantime, we surveyed the somatic mutations for their base mutation patterns, mutation types, and functional outcomes, but did not observe any significant difference between the tumor and normal esophageal mucosal tissues, indicative of no obvious preference.

The mutated genes in ESCC are significantly enriched in cancer-related pathways such as HIPPO, WNT, and NRF2 signaling pathways, which have been implicated in ESCC according to other studies (29–31). In the analysis of mutation signature, Age was the dominant signature in all tissues, consistent with the aged nature of the study cohorts, and this signature was not significantly altered during mucosal cancerization. The proportion of APOBEC signature in tumor tissues was significantly higher than that in normal mucosa. The APOBEC family was a class of gene-editing enzymes that specifically catalyzed the conversion of cytosine in the genome to uracil, participating in the innate immune and antiviral responses of the human body (32). Meanwhile, the APOBEC mutation has also been shown to associate with cancer (32–34). The enrichment of APOBEC mutational signature has also been identified by other studies, and our finding supported that the APOBEC signature could be a potential marker underlying the occurrence and development of ESCC.

Lifestyle factors such as smoking, drinking, and poor eating habits often cause long-term irritation to the entire esophageal mucosa and even the upper aerodigestive tract mucosa, leading to carcinogenesis of the mucosa (35). Such type of irritation could often cause multiple lesions in the esophagus or even multiple primary cancers of the upper aerodigestive tract as found in clinical practice. We attempted to understand the evolutionary and developmental processes of multiple primary tumors and therefore analyzed 8 cases of bifocal esophageal cancer alongside their corresponding mucosa. We found no convergent relationship among the three tissues in 62.5% (5/8) patients, convergence between mucosa and one primary tumor in 12.5% (1/8) patients, and convergence between the two primary tumors in 25% (2/8) patients. These findings reflected tumor lineage diversity in multiple primary esophageal carcinomas, shedding light on the development of ESCC. A high degree of interfocal heterogeneity appears to be common as found in 75.0% (6/8) cases where the two primary tumors are not clustered in the same clade, suggesting that the cancerization processes of different lesions often have a low correlation. In this study, the mode of convergence between the two primary tumors is relatively rare in multiple primary ESCC, in which the cancerization process of lesions might be affected by the shared mutations. Admittedly, the small sample size is a limitation of our study, and more cases are needed to accurately estimate the proportion of different modes and explore other potential patterns.

Due to the ethical requirement and sample availability, we took normal esophageal mucosa from five GC patients who underwent total gastrectomy for comparison with the samples from ESCC patients. We collected all the GC PNM samples > 10 cm far away from the GC lesions and ensured that no lesion was present in the esophagus of these five GC patients. The differences between the tissues from ESCC patients and non-esophageal cancer patients could help pinpoint the molecular mechanisms underlying ESCC. We noticed that some cancer-related genes such as *NOTCH1/2* were mutated in the esophageal PNM of both GC and ESCC, but the mutation frequency in GC was relatively lower than that in ESCC. Interestingly, we found that the mutation frequency of *TP53* in ESCC tumors and PNM was significantly higher than that in the GC group, consistent with the high incidence of *TP53* mutations in ESCC reported by other studies (36). Our finding further supported the role of alterations in *TP53* and its signaling pathway in the carcinogenesis of ESCC. In addition, the *TP53* mutation pattern may predict cancer etiology (17), whereas the most frequent change of G:C>T:A transversion in our cohort is related to tobacco smoking in ESCC, which is consistent with the majority of patients having a smoking history (**Table 1**). Again, our study is restricted by the small sample size, such as the limited number of *TP53* mutations. Further studies with more ESCC and non-ESCC samples are warranted to improve the prediction accuracy, enable more comprehensive analysis, and clearly delineate the unique molecular features of this disease.

In conclusion, we performed a genome-wide analysis of genetic variations in tumor and PNM of ESCC, as well as PNM of non-ESCC controls. Our comparative studies revealed important differences that are related to the carcinogenesis of ESCC. Normal esophageal mucosa showed a high frequency of *NOTCH1/2* mutations. By contrast, gene and chromosomal arm level copy number amplification and chromosomal instability were significantly higher in ESCC tumor samples. Mutated genes in ESCC are enriched in cancer-related pathways, such as HIPPO, WNT, and NRF2 signaling pathways. Using samples from multiple primary esophageal cancers, we conducted phylogenetic analysis and revealed three evolutionary modes from the eight bifocal ESCC patients. In most of the patients, the two primary tumors and the normal esophageal mucosa are all divergent from each other. Finally, the comparison with esophageal PNM samples from non-esophageal gastric cancer patients showed that the frequency of *TP53* mutation was significantly higher in the tissues from ESCC patients. The relatively small sample size is a limitation of this study. Additionally, the follow-up information would be informative to explore the significance of our discoveries, such as patient stratification and prognosis prediction. We plan to address the questions in our following studies.

## Data Availability Statement

The data presented in the study are deposited in the Genome Sequence Archive for Human (GSA-Human) repository, accession number HRA002165.

## Ethics Statement

The studies involving human participants were reviewed and approved by Medical Ethics Committee of Zhejiang Cancer Hospital (Approval No. IRB-2022-154). The patients/participants provided their written informed consent to participate in this study.

## Author Contributions

JLia and YW wrote the manuscript. LC collected specimens and extracted data. JLia and JLiu processed the data analysis. JY, XC, and XW performed WES-related experiments. QC revised the final manuscript. All authors contributed to the article and approved the submitted version.

## Funding

This work was supported by grants from Zhejiang Province Public Welfare Technology Application Research Project (Animal Experiment Project) (No. LGD20H160002), and Medical Health Science and Technology Project of Zhejiang Provincial Health Commission (No. 2020KY083).

## Conflict of Interest

JY, XC, and XW are the employees of Nanjing Geneseeq Technology Inc.

The remaining authors declare that the research was conducted in the absence of any commercial or financial relationships that could be construed as a potential conflict of interest.

## Publisher’s Note

All claims expressed in this article are solely those of the authors and do not necessarily represent those of their affiliated organizations, or those of the publisher, the editors and the reviewers. Any product that may be evaluated in this article, or claim that may be made by its manufacturer, is not guaranteed or endorsed by the publisher.

## Supplementary Material

Supplementary Figure 1 Overview of samples and study design. 18 patients, including 13 ESCC (double-primary: 8, P1-P8; single-primary: 5, P9-P13) and 5 GC patients (P14-P18). One specimen was collected from each primary tumor of ESCC, PNM of ESCC, and PNM of GC. In total, 39 tissue samples were collected and subject to WES for further comparison of their genomic alterations.

Supplementary Figure 2 Overview of mutation types in ESCC PNM and tumor. **(A, B)** show the base mutation patterns in PNM and tumor of ESCC, respectively. The top and bottom panels show the proportions of the changes in all samples and each sample separately. **(C)** Proportions of different SNV types in ESCC PNM and tumor. **(D)** Proportions of inactivation/non-inactivation alterations in ESCC PNM and tumor.

Supplementary Figure 3 Overview of *NOTCH1/2* mutations in ESCC PNM and tumor. **(A, B)** Localizations of different types of SNVs in the NOTCH1 and NOTCH2 proteins as detected in ESCC PNM and tumor. **(C, D)** Proportions of different types of *NOTCH1/2* SNVs in ESCC PNM and tumor.

Supplementary Figure 4 Box plots showing the weights of mutation signatures corresponding to the mutations identified in ESCC PNM and tumor. Note that the APOBEC signature is significantly different between PNM and the tumor.

Supplementary Figure 5 Comparison of GC and ESCC samples. **(A)** Details of genetic alteration type and distribution in the five GC PNM samples. **(B)** Comparison of mutation numbers per sample in ESCC tumor and PNM, as well as GC PNM. **(C)** The proportions of patients carrying mutations in the signaling pathway-related genes between GC and ESCC samples. Note that the frequency of the *TP53* signaling pathway is significantly lower in GC PNM.

Table S1 Baseline characteristics of non-ESCC (gastric cancer) patients enrolled in this study.

Table S2 Mutations shared in PNM and tumor samples of ESCC.

Table S3
*TP53* somatic mutations detected in all ESCC samples.
