# Supplementary figures and images for: Comparative Genomic Analysis Reveals Genetic Variations in Multiple Primary Esophageal Squamous Cell Carcinoma of Chinese Population
Source: Front Oncol. 2022 Apr 20;12:868301. doi: 10.3389/fonc.2022.868301 (PMC9065449; doi:10.3389/fonc.2022.868301)

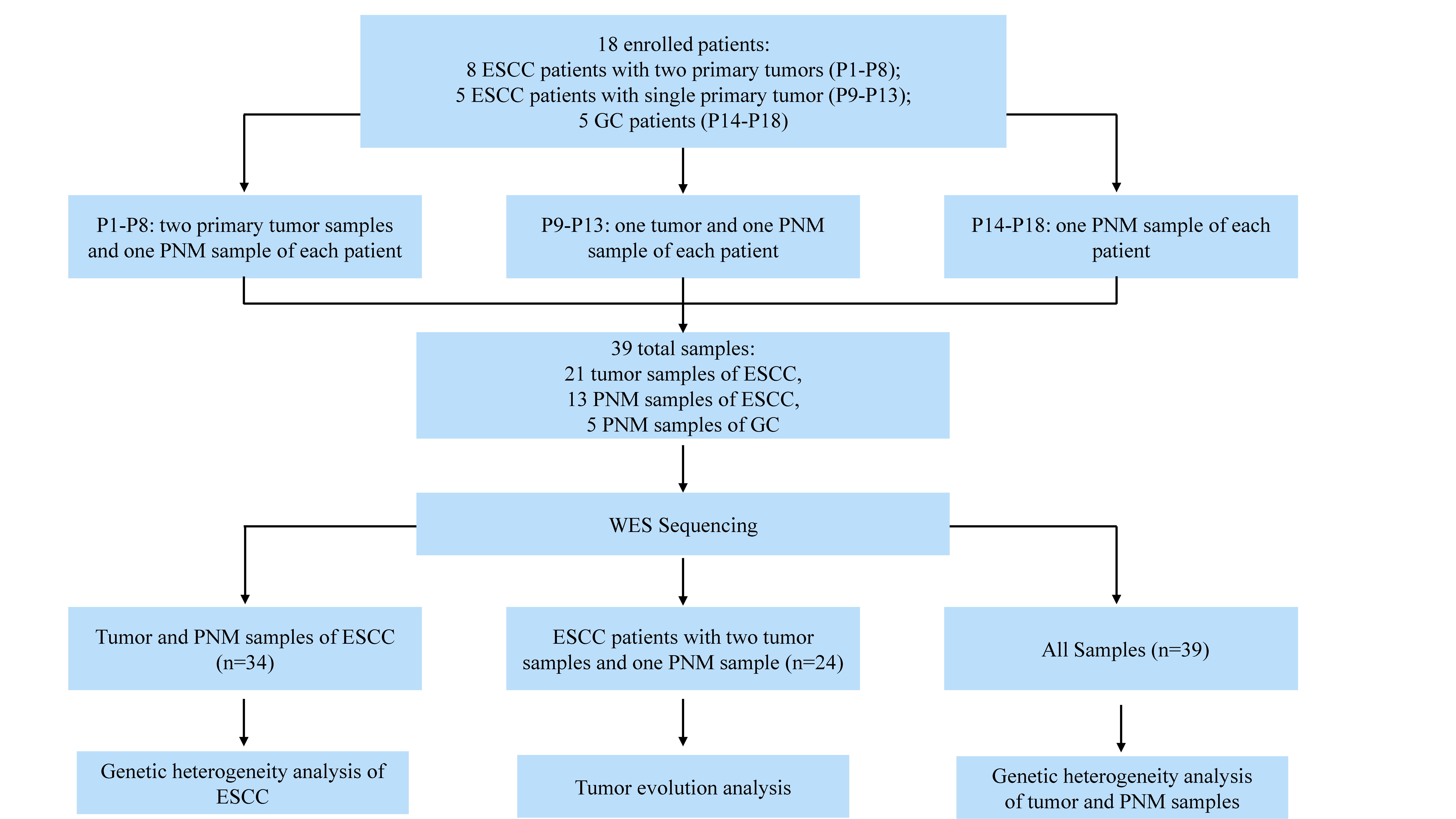

Supplement: Supplementary Figure 1 — Overview of samples and study design. 18 patients, including 13 ESCC (double-primary: 8, P1-P8; single-primary: 5, P9-P13) and 5 GC patients (P14-P18). One specimen was collected from each primary tumor of ESCC, PNM of ESCC, and PNM of GC. In total, 39 tissue samples were collected and subject to WES for further comparison of their genomic alterations. [file Image_1.tiff]

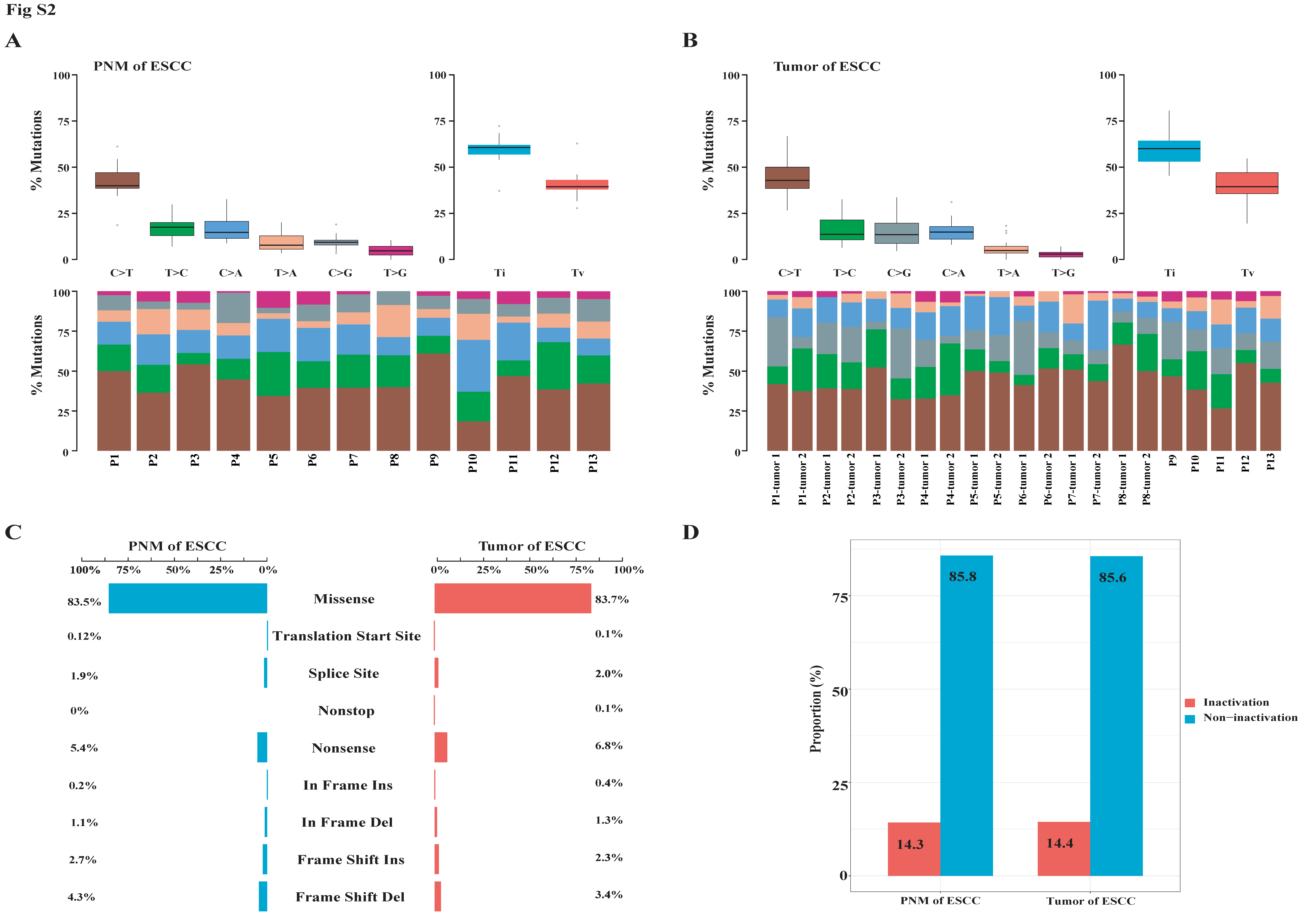

Supplement: Supplementary Figure 2 — Overview of mutation types in ESCC PNM and tumor. (A, B) show the base mutation patterns in PNM and tumor of ESCC, respectively. The top and bottom panels show the proportions of the changes in all samples and each sample separately. (C) Proportions of different SNV types in ESCC PNM and tumor. (D) Proportions of inactivation/non-inactivation alterations in ESCC PNM and tumor. [file Image_2.tif]

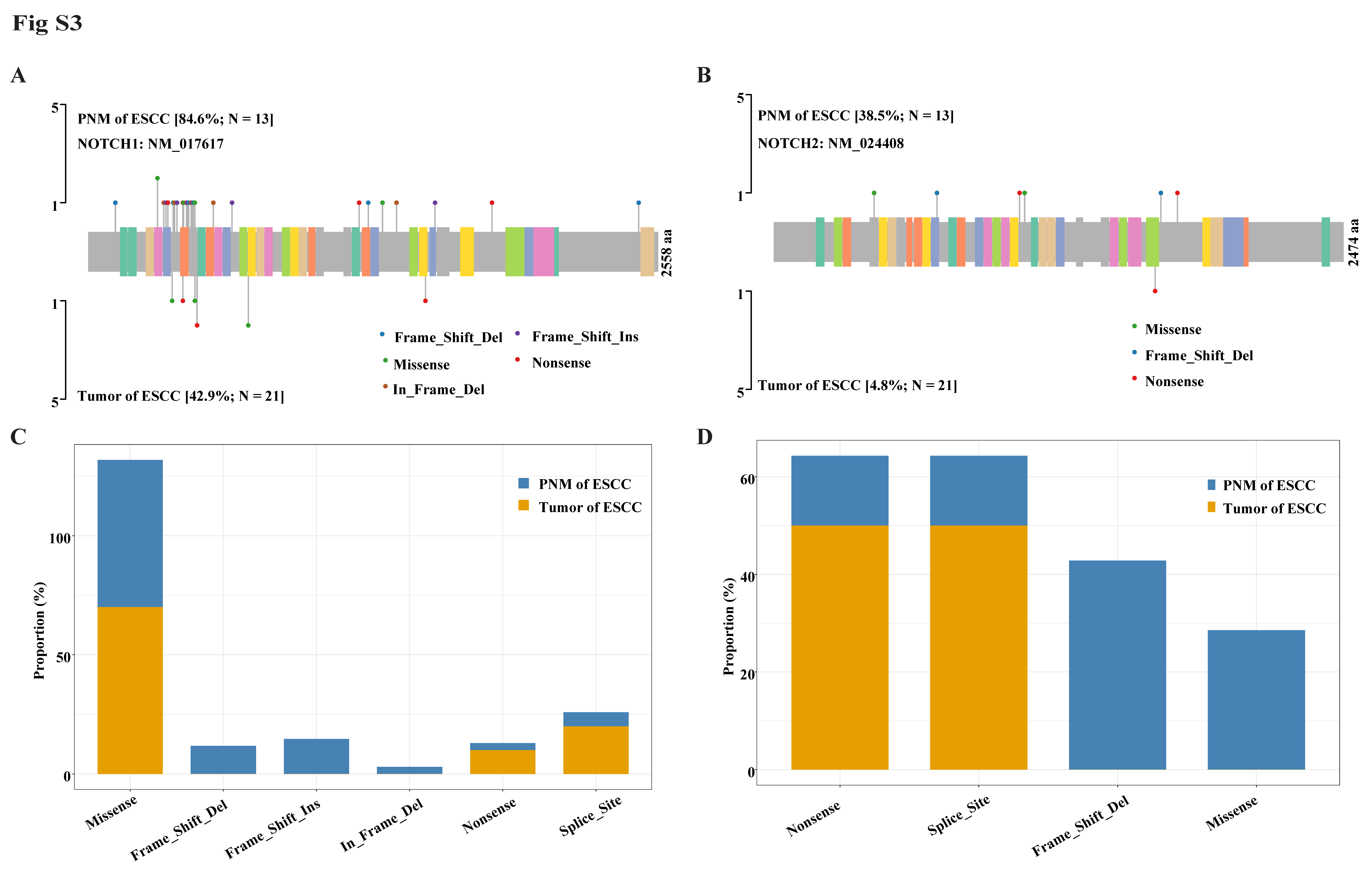

Supplement: Supplementary Figure 3 — Overview of NOTCH1/2 mutations in ESCC PNM and tumor. (A, B) Localizations of different types of SNVs in the NOTCH1 and NOTCH2 proteins as detected in ESCC PNM and tumor. (C, D) Proportions of different types of NOTCH1/2 SNVs in ESCC PNM and tumor. [file Image_3.tif]

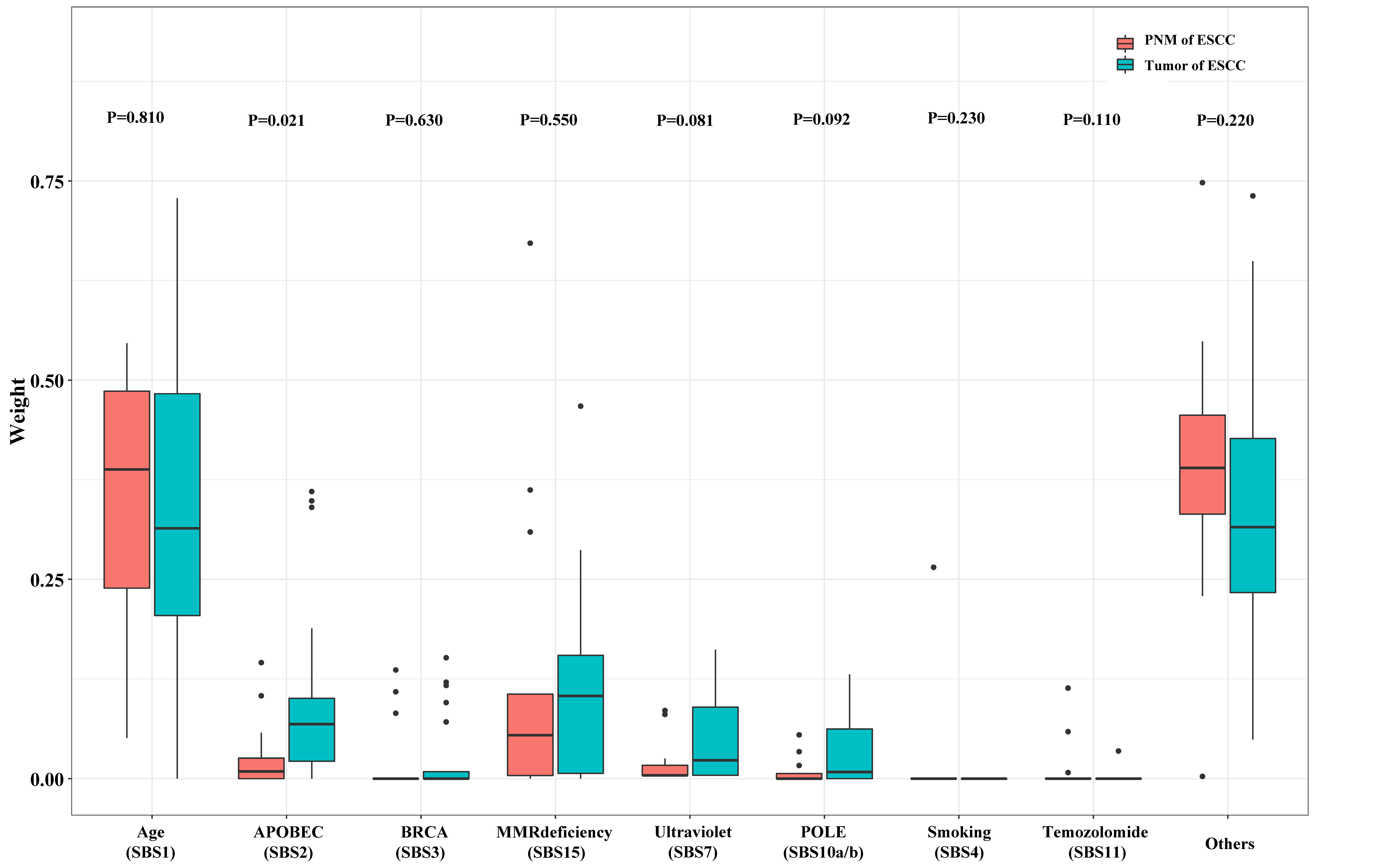

Supplement: Supplementary Figure 4 — Box plots showing the weights of mutation signatures corresponding to the mutations identified in ESCC PNM and tumor. Note that the APOBEC signature is significantly different between PNM and the tumor. [file Image_4.tif]

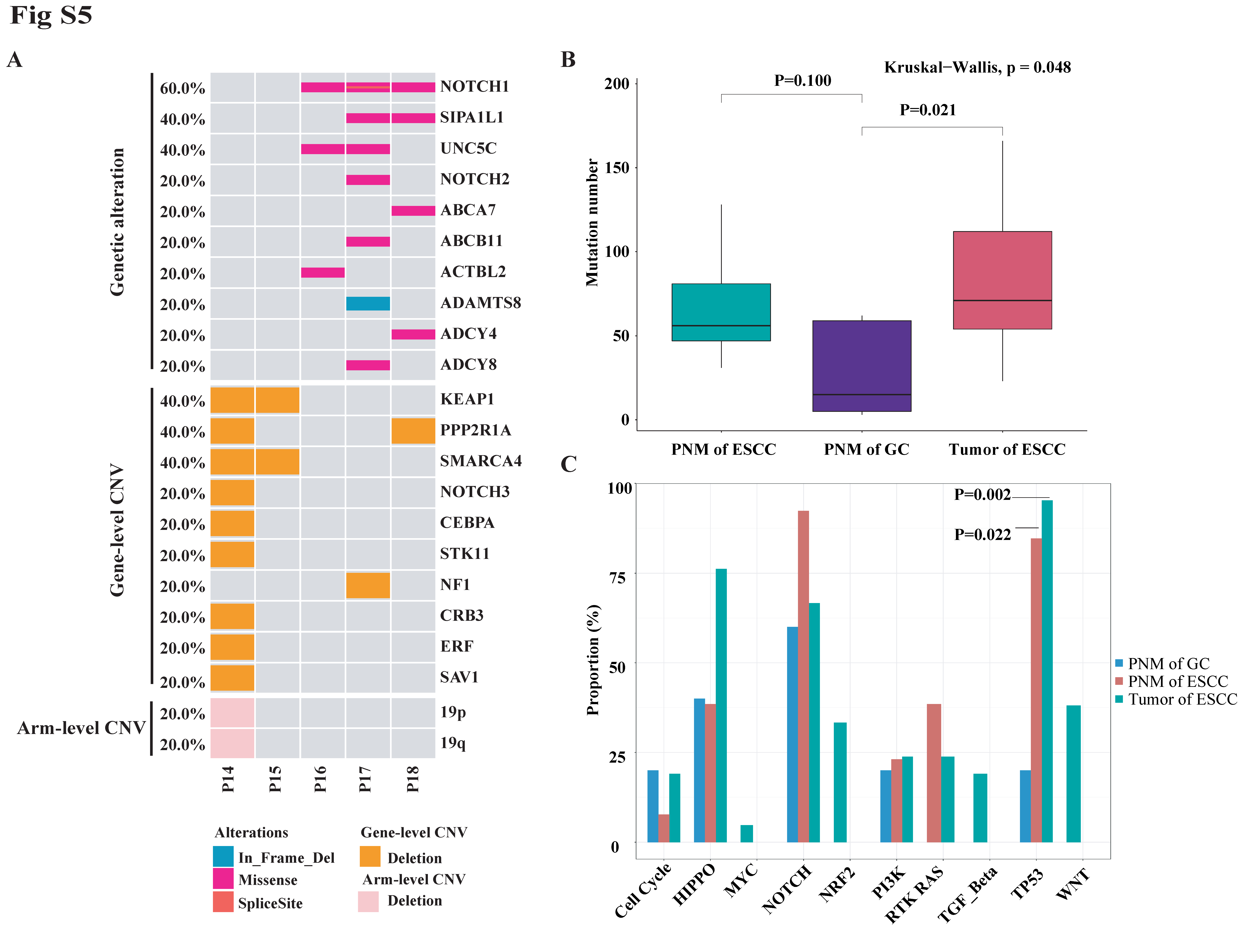

Supplement: Supplementary Figure 5 — Comparison of GC and ESCC samples. (A) Details of genetic alteration type and distribution in the five GC PNM samples. (B) Comparison of mutation numbers per sample in ESCC tumor and PNM, as well as GC PNM. (C) The proportions of patients carrying mutations in the signaling pathway-related genes between GC and ESCC samples. Note that the frequency of the TP53 signaling pathway is significantly lower in GC PNM. [file Image_5.tif]

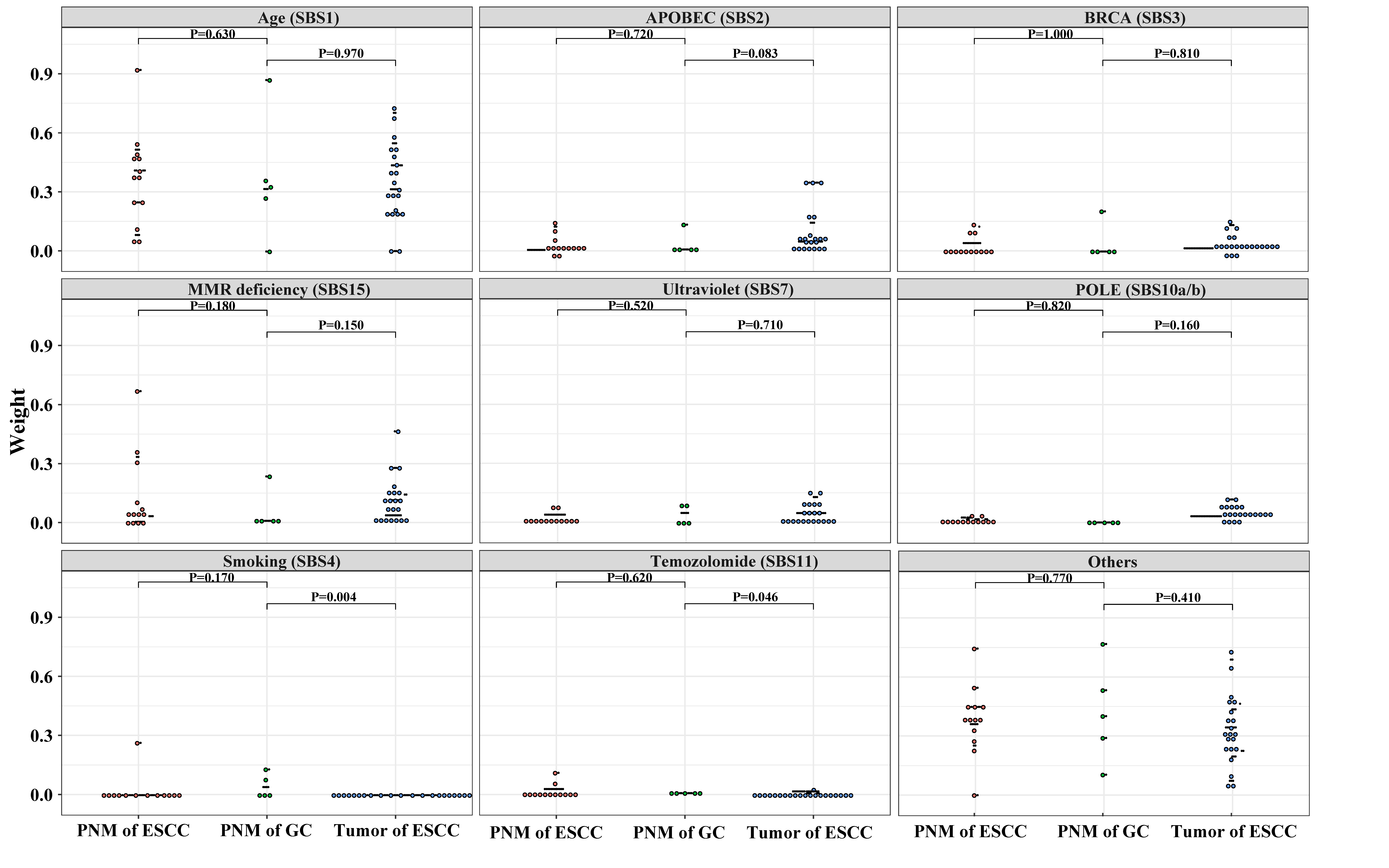

Supplement: Supplementary file 6 [file Image_6.tif]
