# Supplementary material for: Comparative Genomic Analysis Reveals Genetic Variations in Multiple Primary Esophageal Squamous Cell Carcinoma of Chinese Population
Source: Front Oncol. 2022 Apr 20;12:868301. doi: 10.3389/fonc.2022.868301 (PMC9065449; doi:10.3389/fonc.2022.868301)
Supplement: Table S1 — Baseline characteristics of non-ESCC (gastric cancer) patients enrolled in this study. [file Table_1.docx]

Table S1. Baseline characteristics of non-ESCC (gastric cancer) patients enrolled in this study.

| Variables | Number (%) |
| --- | --- |
| Total | 5 (100%) |
| Median age, years (range) | 71 (67-81) |
| Gender |  |
| Male | 5 (100.0%) |
| Female | 0 (0%) |
| Smoking history |  |
| Yes | 4 (80.0%) |
| No | 1 (20.0%) |
| Tumor differentiation |  |
| high | 1 (20.0%) |
| moderate | 2 (40.0%) |
| low | 2 (40.0%) |
| Tumor differentiation |  |
| high | 1 (20.0%) |
| moderate | 1 (20.0%) |
| low | 3 (60.0%) |
| T stage |  |
| T1-3 | 0 (0%) |
| T4 | 5 (100.0%) |
| N stage |  |
| N0 | 2 (40.0%) |
| N1 | 1 (20.0%) |
| N2 | 1 (20.0%) |
| N3 | 1 (20.0%) |
| TNM stage |  |
| I | 0 (0%) |
| II | 2 (40.0%) |
| III | 3 (60.0%) |
